# An extended DeLone and McLean’s model to determine the success factors of e-learning platform

**DOI:** 10.7717/peerj-cs.876

**Published:** 2022-06-23

**Authors:** Raed Shujaa Alotaibi, Saeed M. Alshahrani

**Affiliations:** 1 Department of Computer Science/Shaqra Community College, Shaqra University, Shaqra, Saudi Arabia; 2 Department of Computer Science/College of Computing and Information Technology, Shaqra University, Shaqra, Saudi Arabia

**Keywords:** E-learning platform, DeLone and McLean’s model, Success factors

## Abstract

Due to the COVID-19 pandemic, all Saudi universities have adopted e-learning systems to ensure that educational activities continue. Shaqra University adopted a platform called the Shaqra University e-learning platform. This study aimed to identify the factors contributing to the success of that platform in Shaqra University, based on students’ responses. This research has proposed an extension of well-known DeLone and McLean’s Information Systems Success (D&M ISS) model to check and validate the success factors of the Shaqra University platform. The questionnaire was adopted in this study to collect data from students currently enrolled at Shaqra University. One thousand online links to the questionnaire were randomly distributed among current students enrolled in Shaqra University. The results revealed that the instrument adopted in this study was valid and reliable. Also, the results showed that the model was a good fit for the Saudi context. The proposed factors of instructor’s quality, learner quality, and perceived usefulness positively impacted the e-learning platform. On the other hand, the factors information quality, system quality and service quality had no positive impact on the use of the e-learning platform.

## Introduction

In Saudi Arabia, most universities have recently adopted e-learning choices in their educational activities (Muhammad et al., 2020). According to Ansari et al. (2021), e-learning choices in educational activities were not common among educational institutes. However, after the COVID-19 pandemic, all Saudi institutions began using e-learning systems and platforms to continue delivering educational content to students. Shaqra University adopted the Shaqra University e-learning platform to ensure that education could continue online. Therefore, this study aimed to identify the factors contributing to the success of that platform in Shaqra University, based on students’ responses.

Shaqra University, located in the middle of Saudi Arabia, was established in 2009. It has more than 30,000 students studying in 24 colleges. The components of its e-learning platform are Learning Objects Repository (L.O.R.); a learning management system (Moodle); virtual classrooms; mobile learning; analytic reports; and an ePortfolio (Naseej, 2021).

E-learning has been defined as using modern technology in educational activities (Shakah, Al-Oqaily & Alqudah, 2019). E-learning does not need interactive face-to-face contact between lecturers and students. Some objectives of e-learning include encouraging students’ innovativeness and creativity, improving their skills and improving their productivity (Apriani et al., 2021). According to Nguyen et al. (2020), common terms used in e-learning include computer-based training (C.B.T.), computer-based instruction (C.B.I.), technology-enhanced learning (TEL) and learning platforms.

### E-learning

According to Choudhury & Pattnaik (2020), e-learning is defined as “the transfer of knowledge and skills, in a well-designed course content that has established accreditations, through an electronic media like the Internet, Web 4.0, intranets and extranets”. E-learning is an educational system that provides training and education and it is web-based to disseminate knowledge, information and communication between stakeholders (Cidral et al., 2018). E-learning is being adopted and widely used at all levels in education: universities, secondary schools and primary schools (Hubalovsky, Hubalovska & Musilek, 2019). E-learning enables learners to improve and develop by exploiting its characteristics in educational processes (Choudhury & Pattnaik, 2020). E-learning has advantages such as improving students’ study skills by engaging them ineffective study and allowing students to study anywhere and anytime (Wu et al., 2020). Al-Fraihat et al. (2020) reported that education institutes broadly adopt e-learning. For example, in the U.K., 95% of higher education institutes have already adopted and used learning management systems (L.M.S.) in their educational processes. Smart technology has already impacted teaching methods, learning and education. Consequently, e-learning is expanding quickly due to these technologies and devices’ access to learning resources (Al-Fraihat et al., 2020).

The authors in Gul et al. (2017) have proposed a mind map-based method for improving the students’ learning skills. They concluded that mind map-based methods worked well for both text and block-based languages to improve student’s learning outcomes.

### Theoretical framework

In the past, the study of information systems success was inaccurate and scarce due to the complex nature of the systems (Petter, DeLone & McLean, 2008). In 1992, DeLone and McLean’s Information Systems Success (D&M ISS) model was proposed to determine the success of information systems. They focused on six factors: system quality, information quality; use; user satisfaction, individual impact, and organizational impact (Yakubu & Dasuki, 2018). DeLone and McLean revised the model in 2003 to study researchers’ feedback regarding the weaknesses and strengths of the model. In this phase, they grouped individual and organizational impact as net benefits and added a new factor, service quality, because service is essential in I.S. success. They also divided the use, intention to use and actual use (Yakubu & Dasuki, 2018). According to DeLone & McLean (2003), system quality refers to system performance, technical characteristics and user-friendliness. Service quality refers to the extent of technological staff response and competence. Information quality refers to how the system content is accurate, valid and available. Use is considered the first phase of system success because effective use means full adoption. Users’ satisfaction refers to users’ degree of satisfaction and acceptance of the system. Net benefits are defined as “the perceived individual and organizational impacts on tasks/job performance and efficiency” (Cidral et al., 2018).

**Figure 1:**
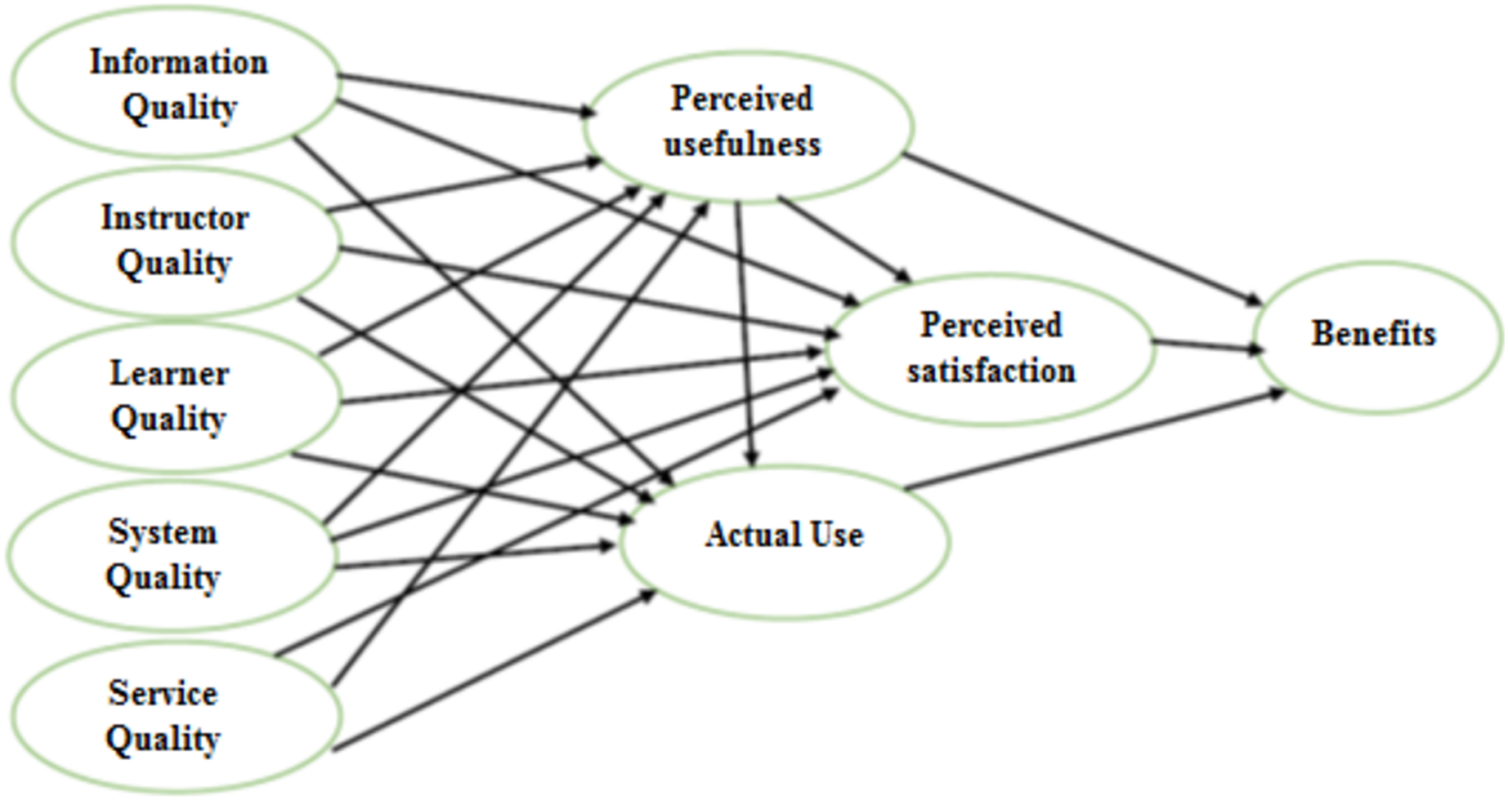
Proposed research model.

This study’s research model was a modified and updated version of the D&M-IS success model (Fig. 1). In addition, three constructs were added: (1) perceived usefulness from the Technology Acceptance Model (T.A.M.); (2) Learner quality; and (3) Instructor quality. Those constructs were adopted in other studies of e-learning, such as those by Cidral et al. (2018), Al-Fraihat et al. (2020), and Ozkan & Koseler (2009).

### Information quality

Information quality is considered an essential factor in assessing the e-learning system’s success. It plays a vital role in the learning process and may cause complex problems if poor information quality (Al-Sabawy, 2013). In the updated DeLone and McLean’s Information Systems Success (D&M ISS) model (DeLone & McLean, 2003), there is a relationship between information quality on the one hand and use and user satisfaction on the other. The study by Rai, Lang & Welker (2002) revealed that information quality was significantly related to use. Those results were consistent with the studies of Halawi, McCarthy & Aronson (2008) in knowledge management systems and Kositanurit, Ngwenyama & Osei-Bryson (2006) in health information systems. In addition, a significant relationship was found between information quality and perceived satisfaction and usefulness (Seddon & Kiew, 1994; Seddon, 1997).

In the e-learning context, some empirical studies have measured the relationship between information quality on the one hand and both use and perceived satisfaction and perceived usefulness on the other. For example, Klobas & McGill (2010) and Eom et al. (2012) found information quality was significantly related to use and satisfaction in L.M.S. Lwoga (2014) found a significant relationship between information quality and perceived usefulness in web-based L.M.S.s. This was consistent with the findings of Chen (2010) in e-learning systems. A recent study by Al-Fraihat et al. (2020) found that information quality positively impacted perceived satisfaction and usefulness of the e-learning system in a U.K. university. Consequently, the following is hypothesized:

H1: Information quality will have a significant positive impact on the perceived usefulness of the Shaqra University e-learning platform.
H2: Information quality will have a significant positive impact on using the Shaqra University e-learning platform.
H3: Information quality will significantly impact the perceived satisfaction with the Shaqra University e-learning platform.

### Instructor quality

Instructor quality has a crucial role in successful e-learning and it has attracted the interest of many researchers in this context (Al-Fraihat et al., 2020). Sun et al. (2008) found a significant positive relationship between instructor response timeliness, instructor attitude toward e-learning, and satisfaction with e-learning at two public universities in Taiwan. Cidral et al. (2018) found a positive relationship between instructor attitudes toward e-learning and satisfaction with e-learning in the Brazilian context. Lwoga (2014) found that instructor quality was significantly related to perceived usefulness and user satisfaction with L.M.S. in Tanzania. Mtebe & Raphael (2018) found that instructor quality significantly impacted learners’ satisfaction with the e-learning system at the University of Dar es Salaam, Tanzania. Park (2009) and Roca & Gagné (2008) found that subjective norms, considered an indicator of instructor quality, were significantly related to usefulness and satisfaction. McGill & Klobas (2009) found a significant positive relationship between instructor norms and L.M.S. utilization. A recent study by Al-Fraihat et al. (2020) found that instructor quality positively impacted perceived usefulness and satisfaction of the e-learning system in a U.K. university. Consequently, the following are hypothesized:

H4: Instructor quality will have a significant positive impact on the perceived usefulness of the Shaqra University e-learning platform.
H5: Instructor quality will have a significant positive impact on using the Shaqra University e-learning platform.
H6: Instructor quality will significantly impact the perceived satisfaction with the Shaqra University e-learning platform.

### Learner quality

According to Al-Fraihat et al. (2020), this construct has been successfully adopted in many studies of e-learning. Some previous research studied the subset of the learner quality construct. Park (2009) and Ong, Lai & Wang (2004) found that the learner’s self-efficacy was significantly related to perceived usefulness. McGill & Klobas (2009) found a significant relationship between learner attitude toward L.M.S. and L.M.S. utilization. In addition, it has been found that a significant relationship existed between student involvement and both use and satisfaction of L.M.S. in an Australian university (Klobas & McGill, 2010). A recent study (Castiblanco Jimenez et al., 2021) found that learners’ computer anxiety significantly influenced perceived usefulness in an e-learning tool designed for E.U. farmers and agricultural entrepreneurs. In studies by Ozkan & Koseler (2009) and Sun et al. (2008), there was a significant relationship between learner quality and perceived satisfaction. A recent study by Al-Fraihat et al. (2020) found that learner quality positively impacted the use and perceived usefulness and satisfaction of the e-learning system in a U.K. university. Consequently, the following are hypothesized:

H7: Learner quality will have a significant positive impact on the perceived usefulness of the Shaqra University e-learning platform.
H8: Learner quality will have a significant positive impact on using the Shaqra University e-learning platform.
H9: Learner quality will significantly impact perceived satisfaction with the Shaqra University e-learning platform.

### System quality

There are relationships between system quality, use, and user satisfaction in the updated DeLone and McLean Information Systems Success (D&M ISS) model (DeLone & McLean, 2003). Some previous studies found a significant positive relationship between system quality and use in the information system context (Halawi, McCarthy & Aronson, 2008; Hsieh & Wang, 2007; Iivari, 2005). It was also found that system quality was significantly related to use in an e-learning context (Balaban, Mu & Divjak, 2013; Garcia-Smith & Effken, 2013; Lin, 2007; Marjanovic, Delić & Lalic, 2016).

Some researchers in Gul et al. (2017) worked on the role of IoT in the education system for improving the quality of education in higher educational institutions. According to them, the paradigm shift of IoT will bring new opportunities for e-learning systems.

Some earlier studies found a positive relationship between system quality and user satisfaction (Halawi, McCarthy & Aronson, 2008; Hsieh & Wang, 2007). It was claimed that users would be satisfied with the e-learning system if it had a technical quality (Hassanzadeh, Kanaani & Elahi, 2012).

Studies by Seddon & Kiew (1994) and Seddon (1997) found that system usefulness will increase if the system quality increases. They also found that system quality is a crucial predictor of usefulness. This is consistent with previous studies by Sabherwal, Jeyaraj & Chowa (2006) and Liaw (2008). A recent study by Al-Fraihat et al. (2020) found that Technical System Quality positively impacted the perceived satisfaction and usefulness of the e-learning system in a U.K. university. Consequently, the following are hypothesized:

H10: System quality will have a significant positive impact on the perceived usefulness of the Shaqra University e-learning platform.
H11: System quality will have a significant positive impact on using the Shaqra University e-learning platform.
H12: System quality will significantly impact the perceived satisfaction with the Shaqra University e-learning platform.

### Service quality

The construct of service quality is essential in the DeLone & McLean model (2003). They assumed relationships between service quality and use and user satisfaction. This construct was used in many information system studies such as Chen & Cheng (2009) who found a relationship between service quality and satisfaction in an online shopping system. It was also found that a significant relationship existed between service quality and use in an e-government system (Wang & Liao, 2008). The results of a study by Ozkan & Koseler (2009) and Roca & Gagné (2008) revealed a significant relationship between service quality and satisfaction in an e-learning context. Previous studies by Al-Sabawy (2013) and Ngai, Poon & Chan (2007) found a significant relationship between perceived usefulness and service quality. A recent study by Al-Fraihat et al. (2020) found that service quality positively impacted perceived satisfaction with the e-learning system in a U.K. university. Consequently, the following are hypothesized:

H13: Service quality will have a significant positive impact on the perceived usefulness of the Shaqra University e-learning platform.
H14: Service quality will have a significant positive impact on using the Shaqra University e-learning platform.
H15: Service quality will significantly impact the perceived satisfaction with the Shaqra University e-learning platform.

### Perceived usefulness PU

Davis (1989) used usefulness as a critical construct in the technology acceptance model (T.A.M.). Davis (1989) defined perceived usefulness as “the degree to which a person believes that using a particular system would enhance his or her job performance.” Previous studies (Roca & Gagné, 2008; Davis, 1989) claimed that acceptance is critical in measuring e-learning systems’ success. For example, the study by Arbaugh (2000) found a positive relationship between the perceived usefulness of the course software and student satisfaction with an Internet-based course. Other studies revealed that perceived usefulness significantly impacted user satisfaction (Al-Sabawy, 2013; Seddon, 1997; Limayem & Cheung, 2008). According to Al-Fraihat et al. (2020), students used the e-learning system if they perceived it was helpful. In the e-learning context, some studies measured the relationship between perceived usefulness and the use of e-learning systems (Islam, 2013; Pituch & Lee, 2006; van Raaij & Schepers, 2008; Sandjojo & Wahyuningrum, 2015; Šumak, Heričko & Pušnik, 2011).

The study by Hwang et al. (2008) found that a relationship between usefulness and net benefits in a Taiwanese hospital and the study by Park et al. (2011) found a significant relationship between usefulness and organizational benefit. Gul et al. (2017) found a significant relationship between usefulness and personal impact. The study results by Hasan, Gholamhosseini & Sarkar (2017) revealed a significant relationship between usefulness and (individual and organizational impact). A recent study by Al-Fraihat et al. (2020) found that P.U. positively impacted perceived satisfaction, benefits, and use of the e-learning system. Consequently, the following are hypothesized:

H16: perceived usefulness will have a significant positive impact on the benefits of the Shaqra University e-learning platform.
H17: perceived usefulness will have a significant positive impact on using the Shaqra University e-learning platform.
H18: perceived usefulness will significantly impact perceived satisfaction with Shaqra University’s e-learning platform.

### Perceived satisfaction

Perceived satisfaction was considered an essential factor in measuring information system success. Based on the DeLone and McLean information systems success model (2003), perceived satisfaction significantly impacted benefits (Al-Fraihat et al., 2020). Hassanzadeh, Kanaani & Elahi (2012) asserted that the system will be used and benefits will be gained if users are satisfied when using the system. Cidral et al. (2018) and Hassanzadeh, Kanaani & Elahi (2012) found a significant relationship between perceived satisfaction and individual impacts. A recent study by Al-Fraihat et al. (2020) found that perceived satisfaction toward the e-learning system had positive benefits for students in a U.K. university. Consequently, the following is hypothesized:

H19: Perceived Satisfaction with the Shaqra University e-learning platform will have a significant positive impact on benefits for students.

### Actual use

The Actual Use construct measures information system success in the DeLone & McLean (2003) model and measures acceptance in the T.A.M. model (Davis, 1989). Previous studies have found that the use of the system was significantly related to its benefits (Garcia-Smith & Effken, 2013; Chen & Tseng, 2012; Hou, 2012). A moderate relationship between using the system and its benefits was found by Petter, DeLone & McLean (2008). The use of e-learning systems in training courses positively influenced net benefits for company employees (Chen & Tseng, 2012). Those results were consistent with those in studies by Halawi, McCarthy & Aronson (2008) and Kositanurit, Ngwenyama & Osei-Bryson (2006). A recent study by Al-Fraihat et al. (2020) found that use has a positive impact on the benefits of the e-learning system in a U.K. university. Consequently, the following is hypothesized:

H20: Actual use of the Shaqra University e-learning platform significantly impacts the system’s benefits.

## Materials and Methods

The approach utilized for the quantitative investigation was an online survey (questionnaire) conducted using Google forms. The questionnaire was designed systematically. First, we have designed the questionnaire through a literature and field survey. Then it was validated by the domain experts from some top academicians who were experts in e-learning platforms. Research on the acceptability of new technology often includes questionnaires to identify elements of influence. Questionnaires disguising the identity of respondents can yield honest and straightforward replies that might be very useful for researchers (Al-Hadidi, 2010). The cover page of the questionnaire opens with an invitation. The aims and purposes of the study are summarized in addition to the moral rights of the participants. Ethical practices ensure that although interviewees are encouraged to react, they are not subjected to objectionable pressure to react, are safeguarded against exploitation and misrepresentation and their privacy is safeguarded (Cavana, Delahaye & Sekaran, 2001; Fink, 2012). This study follows the stringent ethical requirements established by the Shaqra University Research Ethics Committee. Before the study commenced, a request for human ethical approval was filed and granted (Ethics Appl.1603202101). One thousand links to the questionnaire were randomly distributed among current students enrolled in Shaqra University. The participants were asked to answer each question by choosing one option from a five-point Likert scale (5 = strongly Agree, 4 = Agree, 3 = Neutral, 2 = Disagree and 1 = strongly Disagree). Five hundred and eighty-five completed responses were returned.

Questionnaire responses were statistically analyzed. Firstly, demographic factors were summarized using descriptive statistics. The next step was to assess the validity and reliability of the instruments used to ensure, according to Sekaran (2003), that the measurement scale was consistent and reliable.

Unit dimensionality and scale validity were assessed using discriminant and convergent validity of the measures used in this research. The entire model was then examined using Structural Equation Modelling (S.E.M.). S.E.M. has been used with the Smart P.L.S. package utilizing the Partial Least Square (P.L.S.) approach. According to Maqsood et al. (2020), P.L.S. can reduce the residual differences of dependent variables better than S.E.M. Data were analyzed utilizing S.E.M. There are two phases of S.E.M. analysis, according to Gefen, Straub & Boudreau (2000). The first relates to evaluating the measuring model (outer model). The second phase is the structural model (inner model). The findings of the analysis are reported in the next section.

## Results

### Demographic data

The results in Table 1 indicate that most participants (53.7%, *n* = 314) were female while 46.3% were male (*n* = 271). In addition, the majority of participants were studying for a Bachelors’s degree (*n* = 527, 90.1%), while the remainder (*n* = 38, 6.5%) and (*n* = 20, 3.4%) were studying for diplomas and Masters degrees, respectively.

**Table 1:** Demographic data.

Information		Number of participants	Percentage of sample
Gender	Male	271	46.3
	Female	314	53.7
	Total	585	100.0
Education level	Diploma	38	6.5
	Bachelor	527	90.1
	Master	20	3.4
	Total	585	100.0

### Testing the goodness of the measurement model (outer model)

This section describes the instrument testing steps. PLS-SEM was used as a primary methodology to evaluate the model used in the study due to its sophistication—nine structures, 34 indicators, and 20 interactions—and PLS-SEM suits those models (Hair et al., 2010). The calculation and structure of the model were tested using SmartPLS version 3.3.3. As per Wong (2013), it is necessary to adequately assess validity and reliability before testing the path coefficients in the structural model. Therefore, we used a criterion adopted by Wong (2013) (see Table 2).

**Table 2:** Checking reliability and validity. Source: Wong (2013).

Whet to check?	What to look for in SwortPLS?	Where is it in the report?	Is it OK?
Reliability			
Indicator reliability	‘*Outer* loadings’ numbers	PLS->Calculation Results->Outer Loadings	Square each of the outer loadings to find the indicator reliability value. 0.70 or higher is preferred. If it is an exploratory research, 0.4 or higher is acceptable (Hulland, 1999)
Internal consistency reliability	“Reliability” numbers	PLS-> Quality Criteria->Overview	Composite reliability should be 0.7 or higher. if it is an exploratory research, 0.6 or higher is acceptable (Bagozzi & Yi, 1988)
Validity			
Convergent validity	“AVE” numbers	PLS -> Quality Criteria-* Overview	It should be 0.5 or higher (Bagozzi & Yi, 1988)
Discriminant validity	“AVE” numbers and Latent Variable Correlations	PLS->Quality Criteria->Overview (for the AVE number as shown above] PLS->Quality Criteria->Latent Variable Correlations	Fornell & Larcker (1981) suggest that the “square root” of AVE of each latent variable should be greater than the correlations among the latent variables

### Reliability

**Figure 2:**
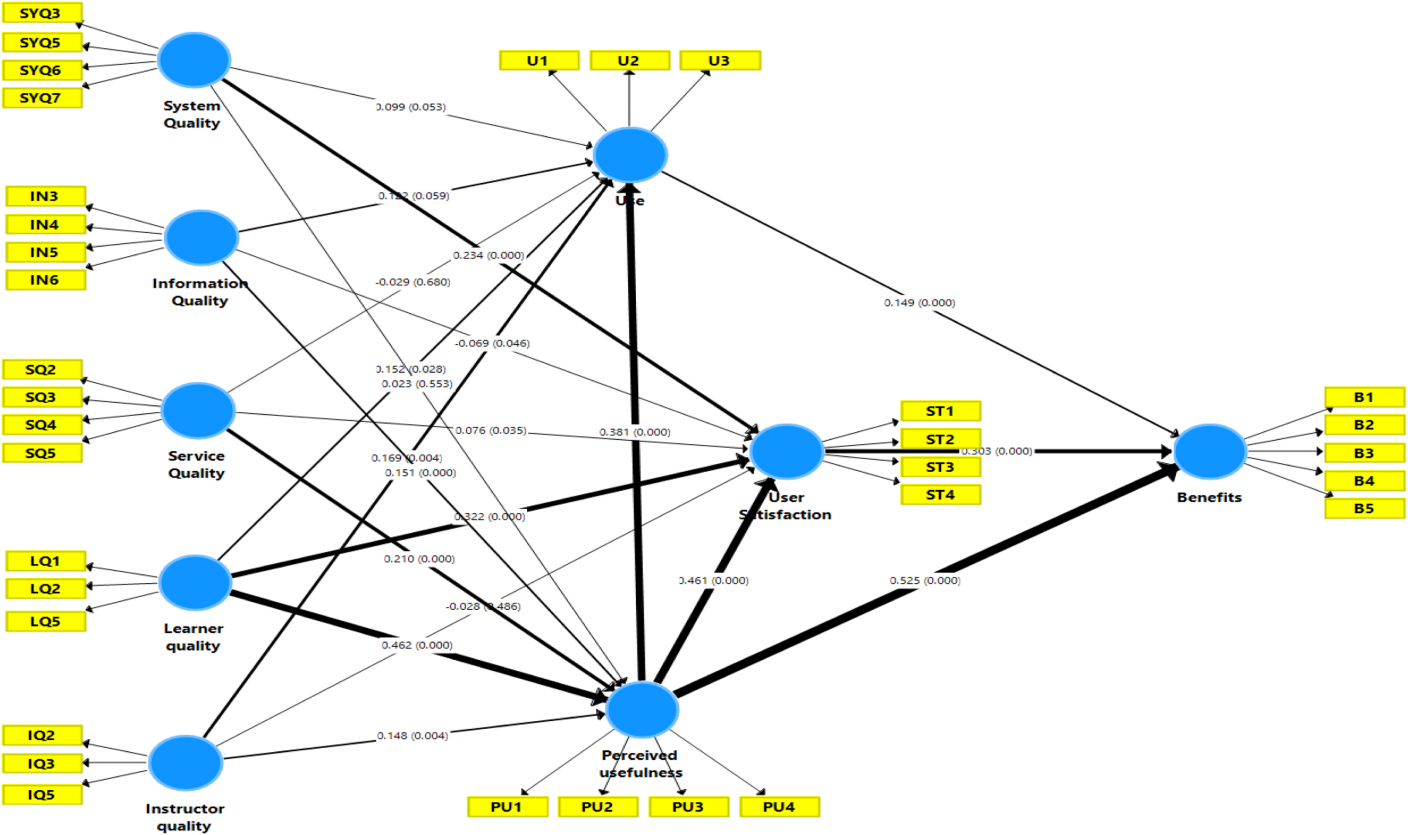
Inner model testing result.

According to Wong (2013) and Sekaran (2003), reliability measures the consistency and stability of the instrument. Thus, as shown in Fig. 2, internal consistency and indicator reliability are required.

### Indicator reliability

The loadings of indicators with respective latent variables are used to assess the reliability of an individual item. To examine the reliability of any individual item, loading and indicator correlations with respective latent variables need to be checked (Wong, 2013). An item’s loadings must be greater than 0.70 to achieve a satisfactory result (Hair et al., 2010). This means that the inclusion of poor indicators may lead to poor fitting in the covariance-based S.E.M. analysis. Accordingly, all items’ loadings were checked, and items with a loading less than 0.70 were deleted.

### Internal consistency reliability

To assess the internal consistency reliability of variables, the composite reliability needs to be checked and the variables’ composite reliability must be at least 0.7 (Wong, 2013). Some researchers argue that Cronbach’s alpha must be for variables that need to be checked in addition to the composite reliability of variables to properly examine the Internal Consistency Reliability of variables, with both required to be higher than 0.7 (Hair et al., 2012; Fornell & Larcker, 1981). In Table 3, both composite reliability and Cronbach’s alpha are at acceptable levels.

**Table 3:** Reliability and validity results.

Construct	Item	Loadings	Cronbach’s alpha	AVE	CR
Benefits	B1	0.933	0.968	0.885	0.975
	B2	0.958			
	B3	0.942			
	B4	0.938			
	B5	0.933			
Information quality	IN3	0.909	0.931	0.829	0.951
	IN4	0.928			
	IN5	0.906			
	IN6	0.899			
Instructor quality	IQ2	0.936	0.928	0.875	0.954
	IQ3	0.934			
	IQ5	0.936			
Learner quality	LQ1	0.941	0.918	0.860	0.948
	LQ2	0.921			
	LQ5	0.920			
Perceived usefulness	PU1	0.946	0.965	0.906	0.975
	PU2	0.957			
	PU3	0.958			
	PU4	0.947			
Service quality	SQ2	0.902	0.931	0.829	0.951
	SQ3	0.911			
	SQ4	0.917			
	SQ5	0.912			
System quality	SYQ3	0.899	0.927	0.821	0.948
	SYQ5	0.902			
	SYQ6	0.892			
	SYQ7	0.932			
Use	U1	0.927	0.925	0.869	0.952
	U2	0.935			
	U3	0.934			
User satisfaction	ST1	0.950	0.964	0.903	0.974
	ST2	0.957			
	ST3	0.949			
	ST4	0.947			

### Validity of scales

To ensure that the model’s reflective measurements were adequate, its discriminant and convergent validity were tested. In addition, the unidimensionality and relevance of the scales were analyzed and the corresponding coefficients were tested. Furthermore, corroborative factor analyses were used to evaluate the merged and discriminating viability of the estimate scales. Finally, the constructions’ validity was evaluated by confirmatory factor analysis (C.F.A.). All of these are discussed below.

### Convergent validity

Convergent validity is used to examine a model’s capacity to explain the indicator’s variance, and the average variance extracted (AVE) value is used to provide evidence for convergent validity (Wong, 2013; Fornell & Larcker, 1981). The AVE value must be at least 0.5 to ensure that convergent validity is at the acceptable level (Wong, 2013). Table 3 shows that all variables’ AVE values are higher than 0.5, which means all variables’ convergent validity is confirmed.

### Discriminant validity

The degree of distinction of objects between structures is discriminant validity (Hair et al., 2014). Furthermore, discriminant validity can be regarded as a statistical metric for examining the level of differentiation between items and constructs. According to Hair et al. (2010), it is better to consider a phenomenon rather than other buildings due to the highly discriminant validity of a building. In this analysis, the validity of the discriminant measurements was checked by the overlap in the variance to ensure the correlation between measurements for each category and the building itself and to make sure that there were no problems in the overload for the measured objects. The discriminant validity of our analysis was measured by two separate metrics, namely, Fornell-Larcker and cross-loading criteria. The Fornell-Larcker criterion is a normative and conservative approach to assessing discrimination (Hair et al., 2016). For this criterion, the independence of a latent variable (equivalent to the square root of the AVE) must be greater than its correspondence with all other latent variables to maintain a discriminant validity. As seen in Table 4 below, the values of the principal diagonal (the self-correlations) are higher than any other value in the same range and column and all latent variables in the model follow this criterion.

**Table 4:** Latent variable correlations.

	B	IN	IQ	LQ	PU	SQ	SYQ	U	ST
B	0.941								
IN	0.825	0.911							
IQ	0.824	0.777	0.935						
LQ	0.868	0.841	0.807	0.927					
PU	0.927	0.846	0.822	0.899	0.952				
SQ	0.835	0.825	0.793	0.822	0.850	0.911			
SYQ	0.823	0.821	0.786	0.810	0.811	0.830	0.906		
U	0.820	0.762	0.755	0.791	0.816	0.738	0.741	0.932	
ST	0.908	0.826	0.802	0.908	0.924	0.848	0.854	0.798	0.951

The cross-loading analysis is the second tool used to assess discriminant validity. Chin (2010) states that the loading value can surpass their loading with other latent variables for each object with its associated latent variable. All elements meet this test, as seen in the cross-loading matrix in Table 5.

**Table 5:** Matrix of cross-loadings.

	B	IN	IQ	LQ	PU	SQ	SYQ	U	ST
B1	0.933	0.770	0.762	0.814	0.864	0.767	0.767	0.784	0.862
B2	0.958	0.778	0.781	0.845	0.895	0.798	0.794	0.767	0.883
B3	0.942	0.785	0.774	0.814	0.877	0.798	0.766	0.767	0.846
B4	0.938	0.762	0.790	0.786	0.852	0.786	0.771	0.762	0.822
B5	0.933	0.784	0.769	0.823	0.874	0.779	0.773	0.779	0.857
IN3	0.761	0.909	0.700	0.786	0.784	0.726	0.747	0.704	0.758
IN4	0.770	0.928	0.697	0.765	0.784	0.759	0.738	0.703	0.756
IN5	0.730	0.906	0.710	0.763	0.756	0.737	0.731	0.692	0.750
IN6	0.742	0.899	0.722	0.750	0.756	0.784	0.778	0.674	0.743
IQ2	0.805	0.742	0.936	0.780	0.790	0.756	0.767	0.734	0.774
IQ3	0.719	0.707	0.934	0.709	0.734	0.720	0.698	0.681	0.698
IQ5	0.783	0.729	0.936	0.772	0.780	0.747	0.737	0.701	0.775
LQ1	0.827	0.773	0.772	0.941	0.851	0.775	0.774	0.766	0.870
LQ2	0.772	0.759	0.748	0.921	0.810	0.750	0.750	0.686	0.820
LQ5	0.813	0.808	0.725	0.920	0.840	0.761	0.728	0.745	0.836
PU1	0.882	0.812	0.766	0.867	0.946	0.802	0.751	0.772	0.872
PU2	0.893	0.799	0.805	0.858	0.957	0.818	0.788	0.779	0.886
PU3	0.887	0.792	0.789	0.850	0.958	0.819	0.784	0.786	0.883
PU4	0.869	0.818	0.770	0.849	0.947	0.798	0.766	0.771	0.878
SQ2	0.781	0.777	0.716	0.787	0.797	0.902	0.792	0.677	0.801
SQ3	0.718	0.737	0.720	0.713	0.733	0.911	0.740	0.640	0.751
SQ4	0.791	0.758	0.728	0.750	0.794	0.917	0.761	0.704	0.798
SQ5	0.747	0.730	0.725	0.740	0.770	0.912	0.728	0.662	0.736
ST1	0.851	0.790	0.758	0.855	0.852	0.829	0.838	0.749	0.950
ST2	0.883	0.801	0.775	0.896	0.895	0.810	0.823	0.784	0.957
ST3	0.867	0.792	0.767	0.853	0.893	0.810	0.783	0.745	0.949
ST4	0.850	0.756	0.750	0.849	0.873	0.776	0.803	0.757	0.947
SYQ3	0.756	0.744	0.715	0.740	0.725	0.749	0.899	0.682	0.750
SYQ5	0.739	0.735	0.720	0.712	0.721	0.752	0.902	0.662	0.747
SYQ6	0.691	0.757	0.690	0.707	0.710	0.713	0.892	0.633	0.753
SYQ7	0.794	0.744	0.725	0.774	0.781	0.793	0.932	0.706	0.841
U1	0.733	0.694	0.681	0.712	0.730	0.665	0.667	0.927	0.722
U2	0.808	0.740	0.731	0.776	0.817	0.719	0.725	0.935	0.783
U3	0.749	0.694	0.697	0.720	0.731	0.676	0.677	0.934	0.725

### Testing the structural model (inner model)

#### Coefficient of determination (R2)

One of the key parameters for the structural model evaluation by PLS-SEM is the Coefficient of Determination, also known as R-Square (R2). More precisely, R2 is the part of variance represented by exogenous variables in the endogenous variable. In Maqsood et al. (2020), the metrics in R2 can be used as a core evaluation of the structural model and, together with the degree of importance of route coefficients, they play a crucial role. The primary objective constructs must have large R2 values as the objective of PLS-SEM is to understand the latent variance of the endogenous variables. Chin (1998) has argued that R2 values under 0.19 are unacceptable. Values of 0.19 to 0.33 are small, 0.33 to 0.67 are mild and 0.67 are strong. The R2 values are, therefore, the basis of the structural model’s quality. In our study’s findings, both R2 values are consistent with Chin (1998) requirements. Table 6 shows that the R2 values for all variables are large, which means that these variables are capable of explaining the model.

**Table 6:** Value of the endogenous latent variables.

	R square	Result
Benefits	0.885	High
Perceived usefulness	0.861	High
Use	0.704	High
User satisfaction	0.904	High

### The effect size (f2)

The effects of R2 variables were calculated by evaluating the effect size (f2) after the assessment of R2 to ascertain whether the effects on the endogenous variable of a given exogenous variable are significant. As recommended by Hair et al. (2014), the following formula was used to calculate f2:

(1) $$f^2={R_{included}^2-R^2_{excluded}\over{1-R^2_{included}}}$$

In both cases, R2 included reflects the condition in which the exogenous latent predictor is a component of the structural model. At the same time, R2 omitted corresponds to values in which the latent exogenous predictor is deleted from the structural model. Ahmad et al. (2020) used the operational concept of multiple regression as a guideline for determining if an exogenous preview component has a big, low, limited, or no impact (f2). This description defines all values below 0.02 as having no effect, values between 0.02 and 0.15 as having a small effect, values between 0.15 and 0.35 as having medium effects and values above 0.35 have a significant impact scale. Table 7 shows the f2 values for this analysis.

**Table 7:** The effect size results.

	B	PU	U	ST
IN		0.035	0.010	0.010
IQ		0.044	0.026	0.002
LQ		0.322	0.012	0.171
PU	0.310		0.068	0.306
SQ		0.069	0.001	0.012
SYQ		0.001	0.008	0.130
U	0.062			
ST	0.112			

### Predictive relevance of the model (Q2)

Predictive validity is another criterion for the consistency evaluation of the structural model (Chin, 2010). Predictive significance is founded on the premise that all indices of an endogenous latent variable must be predictable by the model (Maqsood et al., 2020). The blindfolding protocol was performed to determine Q2 to calculate the commonality of cross-validity (cv-comm) and redundancy of cross-validity (cv-red).

A fixed distance, D, is used to extract data from the data collection through blindfolding procedures. The value of D can be between 5 and 10 (Chin, 2010). The sample size, n, divided by D must be a complete number. The model parameters are then calculated to expect those data volumes to be removed and treated as missing values. However, blindfolding happens only in the case of an endogenous latent variable with reflective dimensions, as in this analysis (Maqsood et al., 2020). Maqsood et al. (2020) supports cross-validity redundancy as it forecasts both the metrics model and the data projection systemic model, which is PLS-SEM-compatible. Bagozzi (1994) argued that the redundancy value of cross-validity above zero (Q2 > 0) would be predictive. On the other hand, a Q2 value below zero shows that the model is not predictable. As seen in Table 8, all endogenous latent variables were greater than negative in the cross-validity redundancies (Q2). This research model therefore has an adequate predictive potential.

**Table 8:** Cross-validity redundancy results.

	Q^2^	Results
B	0.778	Q2 > 0 Explanatory variable provides predictive relevance
PU	0.775	Q2 > 0 Explanatory variable provides predictive relevance
U	0.603	Q2 > 0 Explanatory variable provides predictive relevance
ST	0.811	Q2 > 0 Explanatory variable provides predictive relevance

### The goodness of fit of the model (GoF)

The goodness of fit (GoF) is a global fitness measure (Tenenhaus et al., 2005). The geometric mean indicates that the endogenous variables have an average of R2 and AVE. Its purpose is to analyze the design and calculation of the model, while the actual output is the subject of the model (Chin, 2010; Henseler & Sarstedt, 2013). The calculation method for GoF is as recommended by Hair et al. (2014):

(2) $${GOF} = {\rm \; }\sqrt {\overline {({{R}^2}} *\overline {{AVE}} )}$$

(3) $${GOF} = {\rm \; }\sqrt {\left( {0.838\,*\,0.864} \right)} = 0.851$$

Wetzels, Odekerken-Schröder & Van Oppen (2009) provide the GoF criteria for determining if its values are regarded as small, medium, or large, where values under 0.10 can be considered as small, and values between 0.10 and 0.25 can be considered as medium and values to 0.35 can be considered as significant. In this study, GoF was 0.586, meaning it was sufficiently large enough to ensure the validity of the P.L.S. model.

### Structural model analysis

The relationships between contingent and autonomous buildings derived from calculation models (C.F.A. models) can be explored by scientists on a structural basis. Even so, for the testing of relations between the structures before analysis, it is essential to establish a relationship or a hypothetical model. After real-world findings and a literature review, the hypothetical model was proposed. A path analysis was conducted on the hypothetical model to approximate the coefficients and importance of the relations. Finally, the fit indices for the model were compared to standard fit indices to validate the model’s suitability.

### Collinearity assessment

The systemic model must be assessed before a verdict is reached. In the structural model, collinearity may be a challenge. In more detail, a value of 5 and a more excellent Variance inflation factor (V.I.F.) means that a problem may arise (Maqsood et al., 2020). The findings of the collinearity evaluation are summarized in Table 9. As all V.I.F. values are less than 5, there is no evidence of collinearity between predictor sets.

**Table 9:** Collinearity statistics (V.I.F.).

	Benefits	Perceived usefulness	Use	User satisfaction
Information quality		4.654	4.817	4.817
Instructor quality		3.615	3.772	3.772
Learner quality		4.783	4.324	4.324
Perceived usefulness	4.752		4.215	4.215
Service quality		4.612	4.931	4.931
System quality		4.371	4.375	4.375
Use	3.121			
User satisfaction	4.134			

### Structural model result

The findings of the proposed research model will be discussed in this section *via* the modeling of structural equations (S.E.M.). The literature review shows that using S.E.M.s is common in behavioral science research, especially in IT/IS (Gefen, Straub & Boudreau, 2000). The Smart P.L.S. package using Partial Least Square (P.L.S.) method has applied S.E.M. Fornell, Lorange & Roos (1990) and Wold (1985) argue that P.L.S. is the ideal approach for the exploration of complex models of latent variables.

### Hypothesized structural model

#### Hypothesis testing

The findings obtained during the entire model test were discussed in the forecast portions. First, however, the hypothesized mathematical model and the links between variables must be tested to study the uniqueness of each variable’s contribution to its dependent variable. Hypothesis tests aim to identify which variables add significantly to the interpretation of the dependent variables, together or separately. Figure 1 displays the path coefficients and *p*-values (in parenthesis) for the relationship between the model variables. These will be addressed in-depth in the next paragraph.

### Inner model path coefficient sizes and significance

The structural model, which is well suited to the results, has been studied as the basis for accepting or rejecting the hypothesized relationship, standardized route coefficients and *p*-values. The results shown in Fig. 2 and the theory related to individual model paths are summarized in Table 10. The 5% reliable standard supports assumptions with a value of *p* of the corresponding direction above 0.05 (Hair et al., 2016).

**Table 10:** Hypothesis testing result.

Path (hypothesis)		Std. beta	Std. error	*T*-value	*P*-values	Decision
H1	IN → PU	0.151	0.041	3.659	0.000	Supported**
H2	IN → U	0.122	0.064	1.894	0.059	Not supported
H3	IN → ST	−0.069	0.034	2.004	0.046	Supported*
H4	IQ → PU	0.148	0.052	2.861	0.004	Supported**
H5	IQ → U	0.169	0.059	2.888	0.004	Supported**
H6	IQ → ST	−0.028	0.040	0.697	0.486	Not supported
H7	LQ → PU	0.462	0.048	9.719	0.000	Supported**
H8	LQ → U	0.152	0.069	2.197	0.028	Supported*
H9	LQ → ST	0.322	0.046	6.937	0.000	Supported**
H10	SYQ → PU	0.023	0.038	0.594	0.553	Not supported
H11	SYQ → U	0.099	0.051	1.937	0.053	Not supported
H12	SYQ → ST	0.234	0.035	6.599	0.000	Supported**
H13	SQ → PU	0.210	0.044	4.827	0.000	Supported**
H14	SQ → U	−0.029	0.070	0.413	0.680	Not supported
H15	SQ → ST	0.076	0.036	2.116	0.035	Supported*
H16	PU → B	0.525	0.050	10.483	0.000	Supported**
H17	PU → U	0.381	0.081	4.682	0.000	Supported**
H18	PU → ST	0.461	0.053	8.636	0.000	Supported**
H19	ST → B	0.303	0.045	6.716	0.000	Supported**
H20	U → B	0.149	0.031	4.806	0.000	Supported**

**Notes:**

* Correlation is significant at <0.05.

** Correlation is significant at <0.01.

## Discussion

The empirical results reveal that H1 and H3 are supported. These results indicate that information quality has a significant impact on the perceived usefulness and satisfaction of the e-learning platform. In other words, information quality is considered a determinant for perceived usefulness and satisfaction. In addition, provision of sufficient and required information, information and resources needed, was accessible. The information presented was readily useable. Information was concise and clear. Items that were considered to help students have a valuable and satisfactory platform were that the platform’s structure was well organized into logical and understandable components and that the content was up-to-date. Based on the results, these items may help students complete their tasks quickly and feel satisfied with the e-learning platform. These results are consistent with previous studies (Seddon & Kiew, 1994; Seddon, 1997). These results are also supported by Hassanzadeh, Kanaani & Elahi (2012), who found that information quality significantly impacts satisfaction with e-learning systems in five universities in Iran.

Similarly, these results are supported by a recent study of Al-Fraihat et al. (2020), who found that information quality has a significant positive impact on satisfaction and perceived usefulness of e-learning systems at the University of Warwick in the U.K. Unexpectedly, H2 is rejected. This result indicates that the quality of the information provided to students did not impact their use of the e-learning platform. This may be because using the e-learning platform at Shaqra University is compulsory and the students do not have any choice regarding its use. It may also be the case that students do not need quality information provided by the e-learning platform. If they need any information, they can contact the lecturer for accurate information.

The results revealed that H4 and H5 are supported. These results indicate that instructor quality has a significant impact on the perceived usefulness and the use of the e-learning platform. These results indicate that the Instructor’s quality may help students recognize the usefulness of the e-learning platforms and encourage them to use the e-learning platform effectively. These results are consistent with the study of Lwoga (2014), who found that instructor quality is significantly related to the perceived usefulness of L.M.S. in Tanzania. A recent study by Al-Fraihat et al. (2020) found that instructor quality positively impacted the perceived usefulness of the e-learning system in a U.K. university. McGill & Klobas (2009) found a significant positive relationship between instructor norms and L.M.S. utilization. Unexpectedly, H6 is rejected. This result contradicts some previous studies that found a significant positive relationship between instructor quality and satisfaction with e-learning systems (Cidral et al., 2018; Al-Fraihat et al., 2020; Lwoga, 2014). This may result from students not depending on the e-learning platform to get all resources and submit their tasks (assignments, homework, etc.). This may also indicate that students’ satisfaction can be attributed to factors other than instructor quality. Therefore, instructor quality does not significantly impact students’ satisfaction with the e-learning platform.

The results revealed that H7, H8 and H9 are supported. These results are consistent with many previous studies, such as Chen & Yao (2016) and Üstünel (2016). In addition, these results are consistent with Al-Fraihat et al. (2020), who found that learner quality has a significant impact on perceived usefulness, satisfaction and the use of e-learning systems in a U.K. university. A recent study by Castiblanco Jimenez et al. (2021) found that learners’ computer anxiety significantly influenced perceived usefulness in an e-learning tool designed for E.U. farmers and agricultural entrepreneurs. In addition, the result of a study by McGill & Klobas (2009) revealed that there is a significant relationship between learner attitudes toward L.M.S. and L.M.S. utilization. A significant relationship was also found between student involvement and their use of and satisfaction with L.M.S. in an Australian university (Klobas & McGill, 2010). These results may indicate that learner quality plays a key role in the success of effectively using an e-learning platform. Thus, if a learner has a positive attitude towards using the e-learning platform, has a good experience in using the e-learning platform and can perform tasks, they will use the e-learning platform successfully and effectively. Therefore, Shaqra University should offer training and awareness courses to increase learner quality.

H10 and H11 are statistically rejected. These results indicate that system quality did not impact the usefulness and the use of e-learning systems. These results are inconsistent with some previous studies (Al-Fraihat et al., 2020; Balaban, Mu & Divjak, 2013; Garcia-Smith & Effken, 2013; Lin, 2007; Marjanovic, Delić & Lalic, 2016). However, these results may be because students do not care much about the system’s quality. After all, they must use the e-learning platform regardless of its quality, especially during the Covid-19 pandemic because Shaqra University is shifting all educational activities online *via* the e-learning platform.

Furthermore, students will benefit from the e-learning platform regardless of its quality. The results revealed that H12 is supported. This result is supported by many studies (Cidral et al., 2018; Al-Fraihat et al., 2020; Halawi, McCarthy & Aronson, 2008; Hsieh & Wang, 2007). It may indicate that system quality has a crucial role in student satisfaction. Therefore, Shaqra University should be made aware of this factor and ensure their e-learning platform has a high level of availability, is easy to use, includes interactive features between students and the platform and high-speed information access.

The results revealed that H13 and H15 are supported. These results are consistent with previous studies, such as Al-Fraihat et al. (2020), Al-Sabawy (2013) and Ngai, Poon & Chan (2007). These results indicate that the quality of services provided to students will increase their satisfaction with and the usefulness of using the e-learning platform. In other words, the quality of services provided to students in an e-learning platform, such as proper online assistance and help, good availability of technical support to solve problems, will increase their satisfaction with and the usefulness of the e-learning platform. Unexpectedly, H14 is rejected. This indicates that the quality of the services provided to students does not impact their use of the e-learning platform. This may be because using the e-learning platform at Shaqra University is compulsory. Perhaps students perceive that they do not need a quality service when using the e-learning platform because they must use the Service regardless of its quality. It may also be the case because lecturers help students when they need any help.

H16, H17 and H18 are statistically supported. These results are consistent with previous studies (Al-Fraihat et al., 2020; Islam, 2013; Pituch & Lee, 2006; van Raaij & Schepers, 2008; Sandjojo & Wahyuningrum, 2015; Šumak, Heričko & Pušnik, 2011; Hwang et al., 2008). These results may indicate that perceived usefulness plays a key role in students’ satisfaction in getting benefits from and using the e-learning platform. In other words, if the e-learning platform is useful for students, that will increase the students’ satisfaction, provide them with benefits and promote the use by students of the e-learning platform.

H19 and H20 are supported. These results are consistent with those of Al-Fraihat et al. (2020) who found that perceived satisfaction toward the e-learning system positively benefited students in a U.K. university. It is also consistent with Cidral et al. (2018), who found a significant positive relationship between usage of the e-learning system and the benefits achieved. These results may indicate that students believe the increased use of the e-learning platform will increase the associated benefits. These results may also indicate that student satisfaction plays a key role in achieving the benefits of using the e-learning platform at Shaqra University.

## Conclusions

This study aimed to measure the success factors of using the e-learning platform in Shaqra University based on students’ perspectives. The results revealed that composite reliability and Cronbach’s alpha were at acceptable levels. The results also showed that all variables’ AVE values were higher than 0.5, confirming convergent validity. Furthermore, the R2 values of all variables could explain the model. Finally, the hypothesis testing results showed that except for five hypotheses, all others were supported. These results may contribute to theory by filling the gap in the information systems field, especially in the e-learning area, because it provides the empirical results that measure the success of the e-learning platform in Shaqra University.

Furthermore, these results may be generalizable to all e-learning platforms in Saudi Arabia. Therefore, the proposed model can be used successfully in other countries to measure the success factors of e-learning platforms. By providing a clear picture of the key success factors contributing to the use of the e-learning platform, these results may also contribute practically by assisting Shaqra University to implement an effective e-learning platform.

## Supplemental Information

10.7717/peerj-cs.876/supp-1 Supplemental Information 1 Raw Data.

10.7717/peerj-cs.876/supp-2 Supplemental Information 2 Categorical Data.

10.7717/peerj-cs.876/supp-3 Supplemental Information 3 Questionnaire.

## Additional Information and Declarations

### Data Availability

The following information was supplied regarding data availability: The raw data is available in the Supplemental Files.
