# Supplementary material for: An extended DeLone and McLean’s model to determine the success factors of e-learning platform
Source: PeerJ Comput Sci. 2022 Jun 23;8:e876. doi: 10.7717/peerj-cs.876 (PMC9299266; doi:10.7717/peerj-cs.876)
Supplement: Supplemental Information 2 [file peerj-cs-08-876-s002.pdf]

Questionnaire:

**System Quality SYQ**	
The Shaqra University e-learning platform provides high availability.	SYQ1
The Shaqra University e-learning platform is easy to use.	SYQ2
The Shaqra University e-learning platform user friendly.	SYQ3
The Shaqra University e-learning platform provides interactive features between students and platform	SYQ4
The Shaqra University e-learning platform provides a personalised information presentation.	SYQ5
The Shaqra University e-learning platform has attractive features to appeal to students.	SYQ6
The Shaqra University e-learning platform provides high-speed information access.	SYQ7
In general, I believe that the quality Shaqra University e-learning platform is high.	SYQ8
**Information Quality IN**	
Shaqra University e-learning platform has provided me with sufficient and required information.	IN1
Information and resources needed from Shaqra University e-learning platform are always accessible	IN2
Information from Shaqra University e-learning platform is in a form that is readily useable	IN3
Information in Shaqra University e-learning platform is concise and clear	IN4
The structure of Shaqra University e-learning platform is well organized into logical and understandable components	IN5
The content of Shaqra University e-learning platform is up to date	IN6
I perceive the design of Shaqra University e-learning platform (e.g. fonts, style, colour, images, videos) to be good and meets the quality standards	IN7
**Service Quality SQ**	

There are enough and clear instructions/training about how to use Shaqra University e-learning platform	SQ1
Shaqra University e-learning platform provides proper online assistance and help	SQ2
The IT services staff is available and cooperative when facing an error at Shaqra University e-learning platform	SQ3
The IT services staff understands the specific needs of students	SQ4
I receive a satisfactory and timely response from the IT services staff	SQ5
**Learner Quality LQ**	
I believe it is good to use Shaqra University e-learning platform	LQ1
I have a positive attitude toward using Shaqra University e-learning platform	LQ2
I am not intimidated by using Shaqra University e-learning platform	LQ3
My previous experience with e-learning systems and computer applications helped me in using Shaqra University e-learning platform	LQ4
I am able to perform tasks in Shaqra University e-learning platform successfully	LQ5
**Instructor Quality IQ**	
I use Shaqra University e-learning platform as recommended by my instructors	IQ1
I think an instructor's enthusiasm about using Shaqra University e-learning platform stimulates my desire to learn	IQ2
I receive a prompt response to questions and concerns from my instructors in Shaqra University e-learning platfor	IQ3
I think communicating and interacting with instructors are important and valuable in Shaqra University e-learning platform	IQ4

Generally, my instructors have a positive attitude to the utilization of Shaqra University e-learning platform	IQ5
**Satisfaction ST**	
I am satisfied with the performance of Shaqra University e-learning platform	ST1
I enjoy using Shaqra University e-learning platform in my study	ST2
Shaqra University e-learning platform satisfies my educational needs	ST3
Overall, I am pleased with the experience of using Shaqra University e-learning platform	ST4
**Perceived Usefulness PU**	
Using Shaqra University e-learning platform enables me to accomplish my tasks more quickly	PU1
Using Shaqra University e-learning platform improves my learning performance	PU2
Using Shaqra University e-learning platform helps me learn effectively	PU3
Overall Shaqra University e-learning platform is useful	PU4
**Use U**	
I use Shaqra University e-learning platform frequently	U1
I depend on Shaqra University e-learning platform in my study	U2
I use Shaqra University e-learning platform regularly	U3
On average, I spend a long time on using Shaqra University e-learning platform	U4
**Benefits B**	
The Shaqra University e-learning platform increases my work productivity	B1
The Shaqra University e-learning platform improves the quality of learning.	B2

The Shaqra University e-learning platform facilitates information exchange	B3
The Shaqra University e-learning platform improves collaborative and active learning.	B4
The Shaqra University e-learning platform increased knowledge transfer.	B5
I believe that the Shaqra University e-learning platform helps me to achieve my educational goals..	B6
In general, I believe that Shaqra University e-learning platform is successful and provides me more knowledge for my studies.	B7
