# Supplementary material for: An extended DeLone and McLean’s model to determine the success factors of e-learning platform
Source: PeerJ Comput Sci. 2022 Jun 23;8:e876. doi: 10.7717/peerj-cs.876 (PMC9299266; doi:10.7717/peerj-cs.876)
Supplement: Supplemental Information 3 [file peerj-cs-08-876-s003.pdf]

Dear Participant,

This research aims to measure the success factors of using e-learning platform in Shaqra University based on Students' perspectives. The results of this research will help decision makers in the Shaqra University implement an effective e-learning platform.

Please participate in this survey and your answers will help the researchers analyse the data and will assist researchers to serve the Saudi society. I highly appreciate your time answering all the questions. Completion of this questionnaire will be take around 35-40 minutes. Please read the instructions and complete the questionnaire.

This questionnaire is completely anonymous. Your participation in this study is not-compulsory and you may discontinue your participation any time without explanations or fear of reprisal. Your information will be protected and only used to achieve the research requirements. The researcher has applied for ethical clearance from research ethics committee in college of computing and information technology at Shaqra University (Ref: Ethics Appl.1603202101) to make sure that this research will be conducted in the right way. Your consent to participate in this research will be implied by reading the information, completing and returning the questionnaire.

Please do not hesitate to contact me if you have any questions and thank you so much in advance for your participation.

Regards

Dr. Raed Alotaibi

Alhafi@su.edu.sa

Dr. Saeed Alshahrani

salshahrani@su.edu.sa

Questionnaire:

**System Quality SYQ**	
The Shaqra University e-learning platform provides high availability.	SYQ1
The Shaqra University e-learning platform is easy to use.	SYQ2
The Shaqra University e-learning platform user friendly.	SYQ3
The Shaqra University e-learning platform provides interactive features between students and platform	SYQ4
The Shaqra University e-learning platform provides a personalised information presentation.	SYQ5
The Shaqra University e-learning platform has attractive features to appeal to students.	SYQ6
The Shaqra University e-learning platform provides high-speed information access.	SYQ7
In general, I believe that the quality Shaqra University e-learning platform is high.	SYQ8
**Information Quality IN**	
Shaqra University e-learning platform has provided me with sufficient and required information.	IN1
Information and resources needed from Shaqra University e-learning platform are always accessible	IN2
Information from Shaqra University e-learning platform is in a form that is readily useable	IN3
Information in Shaqra University e-learning platform is concise and clear	IN4
The structure of Shaqra University e-learning platform is well organized into logical and understandable components	IN5
The content of Shaqra University e-learning platform is up to date	IN6
I perceive the design of Shaqra University e-learning platform (e.g. fonts, style, colour, images, videos) to be good and meets the quality standards	IN7
**Service Quality SQ**	

There are enough and clear instructions/training about how to use Shaqra University e-learning platform	SQ1
Shaqra University e-learning platform provides proper online assistance and help	SQ2
The IT services staff is available and cooperative when facing an error at Shaqra University e-learning platform	SQ3
The IT services staff understands the specific needs of students	SQ4
I receive a satisfactory and timely response from the IT services staff	SQ5
**Learner Quality LQ**	
I believe it is good to use Shaqra University e-learning platform	LQ1
I have a positive attitude toward using Shaqra University e-learning platform	LQ2
I am not intimidated by using Shaqra University e-learning platform	LQ3
My previous experience with e-learning systems and computer applications helped me in using Shaqra University e-learning platform	LQ4
I am able to perform tasks in Shaqra University e-learning platform successfully	LQ5
**Instructor Quality IQ**	
I use Shaqra University e-learning platform as recommended by my instructors	IQ1
I think an instructor's enthusiasm about using Shaqra University e-learning platform stimulates my desire to learn	IQ2
I receive a prompt response to questions and concerns from my instructors in Shaqra University e-learning platfor	IQ3
I think communicating and interacting with instructors are important and valuable in Shaqra University e-learning platform	IQ4

Generally, my instructors have a positive attitude to the utilization of Shaqra University e-learning platform	IQ5
**Satisfaction ST**	
I am satisfied with the performance of Shaqra University e-learning platform	ST1
I enjoy using Shaqra University e-learning platform in my study	ST2
Shaqra University e-learning platform satisfies my educational needs	ST3
Overall, I am pleased with the experience of using Shaqra University e-learning platform	ST4
**Perceived Usefulness PU**	
Using Shaqra University e-learning platform enables me to accomplish my tasks more quickly	PU1
Using Shaqra University e-learning platform improves my learning performance	PU2
Using Shaqra University e-learning platform helps me learn effectively	PU3
Overall Shaqra University e-learning platform is useful	PU4
**Use U**	
I use Shaqra University e-learning platform frequently	U1
I depend on Shaqra University e-learning platform in my study	U2
I use Shaqra University e-learning platform regularly	U3
On average, I spend a long time on using Shaqra University e-learning platform	U4
**Benefits B**	
The Shaqra University e-learning platform increases my work productivity	B1
The Shaqra University e-learning platform improves the quality of learning.	B2

The Shaqra University e-learning platform facilitates information exchange	B3
The Shaqra University e-learning platform improves collaborative and active learning.	B4
The Shaqra University e-learning platform increased knowledge transfer.	B5
I believe that the Shaqra University e-learning platform helps me to achieve my educational goals..	B6
In general, I believe that Shaqra University e-learning platform is successful and provides me more knowledge for my studies.	B7
